# Machine Learning to Predict Enzyme–Substrate Interactions in Elucidation of Synthesis Pathways: A Review

**DOI:** 10.3390/metabo14030154

**Published:** 2024-03-07

**Authors:** Luis F. Salas-Nuñez, Alvaro Barrera-Ocampo, Paola A. Caicedo, Natalie Cortes, Edison H. Osorio, Maria F. Villegas-Torres, Andres F. González Barrios

**Affiliations:** 1 Grupo de Diseño de Productos y Procesos (GDPP), Department of Chemical and Food Engineering, Universidad de los Andes, Bogotá 111711, Colombia; lf.salas@uniandes.edu.co; 2 Grupo Natura, Facultad de Ingeniería, Diseño y Ciencias Aplicadas, Departamento de Ciencias Farmacéuticas y Químicas, Universidad ICESI, Calle 18 No. 122-135, Cali 760031, Colombia; aabarrera@icesi.edu.co; 3 Grupo Natura, Facultad de Ingeniería, Diseño y Ciencias Aplicadas, Departamento de Ciencias Biológicas, Bioprocesos y Biotecnología, Universidad ICESI, Calle 18 No. 122-135, Cali 760031, Colombia; pacaicedo@icesi.edu.co; 4 Grupo de Investigación en Química Bioorgánica y Sistemas Moleculares (QBOSMO), Faculty of Natural Sciences and Mathematics, Universidad de Ibagué, Ibagué 730002, Colombia; natalie.cortes@unibague.edu.co (N.C.); edison.osorio@unibague.edu.co (E.H.O.); 5 Centro de Investigaciones Microbiológicas (CIMIC), Department of Biological Sciences, Universidad de los Andes, Bogotá 111711, Colombia; m.f.villegastorres@uniandes.edu.co

**Keywords:** enzyme–substrate interaction, artificial intelligence, synthesis routes, enzyme classification, molecular descriptors, training data, computational studies

## Abstract

Enzyme–substrate interactions play a fundamental role in elucidating synthesis pathways and synthetic biology, as they allow for the understanding of important aspects of a reaction. Establishing the interaction experimentally is a slow and costly process, which is why this problem has been addressed using computational methods such as molecular dynamics, molecular docking, and Monte Carlo simulations. Nevertheless, this type of method tends to be computationally slow when dealing with a large search space. Therefore, in recent years, methods based on artificial intelligence, such as support vector machines, neural networks, or decision trees, have been implemented, significantly reducing the computing time and covering vast search spaces. These methods significantly reduce the computation time and cover broad search spaces, rapidly reducing the number of interacting candidates, as they allow repetitive processes to be automated and patterns to be extracted, are adaptable, and have the capacity to handle large amounts of data. This article analyzes these artificial intelligence-based approaches, presenting their common structure, advantages, disadvantages, limitations, challenges, and future perspectives.

## 1. Introduction

The elucidation of de novo biosynthetic pathways from existing metabolic pathways for the production of high-value compounds is an important field of study for synthetic biology and metabolic engineering [1,2]. For this elucidation, it is necessary to understand enzyme–substrate interactions, which allows us to establish the specificity or promiscuity of a reaction.

However, discovering enzyme–substrate interactions is a complex and costly process, especially when there is no previous bibliographic information that establishes a relationship [3]. Fortunately, artificial intelligence (AI) has proven to be a powerful tool in this regard, as it allows for the establishment of relationships based on patterns. Through the use of machine learning algorithms, there is the potential to predict enzyme–substrate interactions and accelerate the elucidation process of the biosynthetic pathway [4,5,6,7].

One of the most widely used approaches in the prediction of the enzyme–substrate interactions is the use of discriminative machine learning models, such as models based on support vector machines, neural networks, and decision trees [5]. These models are trained using data sets of enzymes that have interactions with the substrate or that catalyze identical reactions with other substrates; these contain information about the amino acid sequences and/or three-dimensional structures of enzymes, and we can also add molecular characteristics of the substrates. Through machine learning, these models can identify patterns and relationships between features and predict enzyme–substrate interactions [7]. This provides a novel and promising perspective for the production of compounds of interest, as it allows the exploration of enzyme–substrate combinations that might not have been previously considered or have not been previously reported.

In this review, we delve into the essential procedures for discerning enzyme–substrate interactions through discriminative machine learning models. The involved process is outlined in the graphical abstract, and we will elaborate on each of the steps in the following paragraphs.

## 2. Fundamentals of Artificial Intelligence

Artificial intelligence (AI) has become increasingly important in our current society as a field of study and development [8] It has permeated nearly every field of human knowledge, showing good performance. In the field of synthetic biology and metabolic engineering, it has had a significant impact on the prediction of enzyme–substrate interactions for pathway elucidation.

Machine learning is one of the fundamental elements of artificial intelligence [9]. Its principle is that machines can learn from the data provided to develop models, policies, and functions that improve their ability to process, analyze, and understand information [8,9]. A common machine learning technique is neural networks, which are composed of interconnected layers of nodes and are applied to process and analyze large amounts of data [10,11]. This ability to process, analyze, and understand large data sets is what makes machine learning a technique used to predict enzyme–substrate interactions, as it allows for the analysis of large chemical, physical, and topological data sets of substrates, as well as the structural and sequential characteristics of enzymes.

Other fundamental elements of artificial intelligence are logic and reasoning, enabling AI systems to make logical decisions and solve problems efficiently. Logic provides a formal and mathematical framework for knowledge representation and manipulation, enabling AI systems to make inferences and decisions based on rules and algorithms [9,12]. Reasoning refers to the mental process of inferring conclusions from premises or available information [13]. In rule-based artificial intelligence, logical rules are used to represent the knowledge of a system and guide its reasoning and decision-making process [14]. In addition to rule-based logic, there are other forms of reasoning, such as probabilistic reasoning and constraint-based reasoning. Probabilistic reasoning uses probability theory to make inferences and decisions in uncertain situations or with incomplete information, while constraint-based reasoning focuses on problem solving by specifying constraints and searching for solutions that satisfy those constraints [15,16]. A technique that uses this principle is decision trees, which consist of a map of the possible outcomes of a series of related decisions [17]. This approach allows for the addition of information provided by an expert on the subject, thus narrowing the search space and reducing processing time. In this way, the application of logic and reasoning will allow the addition of minimum AI model characteristics that an enzyme or substrate should meet, which can come from our knowledge of the reaction type, thus reducing the complexity of the problem.

Artificial intelligence (AI) models aimed at predicting enzyme–substrate interactions have been applied to various classes of enzymes, including nitrilases, thioesterases, oxidoreductases, and dehydrogenases, among others [5,18,19]. In each instance, performance exceeding 70% was achieved, which was also experimentally validated. These results indicate that the algorithms maintain consistent performance across varied experimental conditions. It is essential to highlight that, in many cases, a significantly extensive search space is efficiently reduced by the algorithm to a few candidates that can be subjected to experimental tests. This ability of AI models to address specific enzymatic challenges in diverse contexts underscores their general utility and potential impact on enzymatic research and biotechnological applications.

In recent years, the development of general models for predicting enzyme–substrate interactions has been investigated; however, it has been observed that these models exhibit inferior performance compared to those designed for specific sets of enzymes. In particular, Kroll et al. point out that their model shows deficiencies when faced with substrates that were not included in the training phase [20]. These findings highlight the importance of considering the experimental conditions of each enzymatic space during the training process, as the generalization of models may be limited.

## 3. Training Data—Data Set

Training data play a fundamental role in the success of AI algorithms, as they provide a set of cases or instances to the model to teach it to perform a specific task [21,22]. These data consist of a combination of expected inputs and outputs, so that the model learns to associate the inputs with their respective correct outputs from a training process through functions that capture the dynamics of the system; this process is called training [22,23]. The objective is to teach systems to recognize patterns, make predictions, and make decisions based on real data. It is important to note that the quality and quantity of training data are critical factors that can significantly influence the performance, selection, and accuracy of AI models.

The collection and preparation of training data can be a laborious and demanding process. It is essential to ensure that the data are sufficient, representative, relevant, unrelated, and diverse, as this will allow the model to capture the complexity (generalization) of the scenarios in which AI will be used. Additionally, it is crucial to have the data correctly labeled and annotated so that the models can learn effectively [21].

It is relevant to mention that the training data, in the vast majority of cases, must be balanced, that is, the number of cases or instances of each class that we are going to pass to the model to train it must be similar; if there is a significant disproportion in the number of examples for each class or category, the model can be biased towards the majority classes [21,23,24]. This means that the model may have difficulty recognizing and learning patterns in minority classes, resulting in lower accuracy and performance for those classes [25]. A balanced data set provides a fair and accurate representation for the model, giving appropriate weight to each parameter, although it is not always possible to achieve this representation due to limited data, or reality is not represented [26]. This allows the model to learn and generalize better in new and unknown situations during training. In cases where the data set cannot be symmetrical, data augmentation methods, resampling, or specialized AI methods should be considered.

The availability of training data can vary depending on the domain and the specific problem being addressed [27]. However, in the case of enzymes and substrates, they are more specialized domains where data collection is expensive or complex, availability is limited, the existence of curated data is low, and they have complex characteristics such as the three-dimensional structure of an enzyme [6]. Data for enzymes and substrates are often sparse due to difficulties in collection and experimentation. This can affect the ability of AI models to learn and generalize correctly [4,6]. One possible strategy is to take advantage of public databases containing information on enzymes and substrates, allowing us to create our own training data set [28]. These public databases, such as UniProt, PubChem, KEGG, and Protein Data Bank, can provide valuable data for training AI models [20,29].

The information provided by each database can vary significantly. For instance, UniProt focuses on providing data about the amino acid sequences of enzymes and their functions, while PDB and MetaCyc offer information on the three-dimensional structure of biomolecules and metabolic pathways, respectively [30,31,32,33]. Some databases even encompass genomic information and provide software and tools for sequence analysis and visualization of biological pathways and reactions. The availability and accuracy of these tools can influence the utility of each database. Additionally, databases differ in terms of their data sources and the formats in which data can be exported. Some databases are specific to individual organisms or species, while others cover information about multiple organisms. In addition, it is important to foster collaboration among the scientific community and share data sets to boost research in the field of enzymes. Table 1 summarizes the characteristics of the information provided by each database.

We consider this step, the selection of training data for the use of AI in enzyme–substrate prediction, to be the most important, as enzymes and substrates can exhibit a great variability in terms of sequence, structure, and function. Additionally, enzymes can have subtle similarities and differences, making it difficult to generalize and identify precise patterns.

## 4. Characterization of Enzyme–Substrate Interactions

Characteristics are distinctive qualities or traits that describe something, be it an object, a set of objects, a place, or a situation, and distinguish it from a set of similar data [38,39]. Feature extraction is a crucial step in training artificial intelligence models [40,41]. It consists of identifying and selecting the most relevant characteristics of a training data set to effectively represent the patterns and relationships present in the data. These extracted features are then used as inputs to the artificial intelligence model [24].

These characteristics can be grouped based on statistics, frequency, spatial, temporal, and domain. The selection of the appropriate features depends on the problem being addressed and the type of data available [40]. In the field of enzyme–substrate interactions, these characteristics are known as descriptors, which can be related to both molecular aspects and biochemistry, as well as the attributes used in learning.

**Figure 1:**
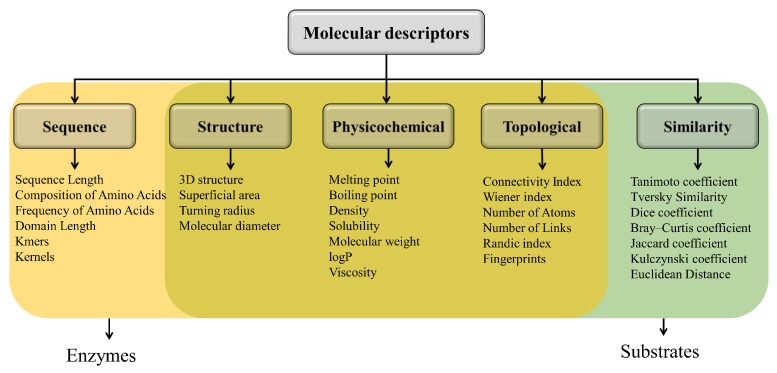
Classification of molecular descriptors for enzymes and substrates. Some descriptors are mentioned in each type and grouped by typical usage. From left to right, typical usage for enzymes; from right to left, typical usage for substrates.

Molecular descriptors are numerical or symbolic representations of molecules that are used to describe their structural, chemical, or physical characteristics [42,43]. In the context of enzymes and substrates, molecular descriptors play a crucial role in predicting enzyme–substrate interactions and in elucidating synthetic pathways by capturing different aspects of enzymes and substrates, providing valuable information to understand their characteristics and properties. The descriptors can be grouped into five types: molecular structure, amino acid sequence, physicochemical, molecular topology, and similarity (Figure 1) [18]. The sequence (amino acid) and structure descriptors are more commonly used in enzymes because they provide genetic information and information about the tertiary structure of these molecules. On the other hand, similarity and topology descriptors make little sense for enzymes due to their large size. For example, the Tanimoto similarity between two enzymes is a less relevant descriptor for machine learning, given the diversity of structures and functions they exhibit [5,18,19,44,45,46,47,48]. The similarity and topology descriptors are more commonly used in substrates, as they allow us to obtain information about their molecular shape, connectivity, and similarity to other compounds. In the case of substrates, sequence and structure descriptors are less relevant because these are small molecules, so it is more convenient to consider the SMILE [18,20,49,50,51]. Physicochemical descriptors are employed for both enzymes and substrates, as they offer valuable information about properties such as electric charges, solubility, and other characteristics relevant to machine learning [5,18,19,20,44,45,46,47,48,49,50,51]. Table 2 presents an overview of the frequently used categories of descriptors for enzymes and substrates, showcasing specific examples of approaches within each category. This comprehensive display serves as a valuable reference for understanding the diverse methodologies employed in enzyme and substrate analysis.

In a study to predict the substrate range of bacterial nitrilases, which catalyze the hydrolysis of nitrile compounds to the corresponding carboxylic acids and ammonia, sequence- and structure-based annotation approaches were used together with specific experimental activity data and physicochemical properties of proteins and ligands with various machine learning models, obtaining performances around 82% [18]. Amin used sequence- and structure-based descriptors to create structural motifs of a few evolutionarily important residues in the training enzymes, and these motifs investigate local evolutionary and geometric similarities in other protein structures to detect functional similarities, testing the predictions experimentally and obtaining good precision [19]. Other articles used sequence alignments and kernels to train intelligence models that discriminate the function of a substrate [5,44,46].

**Figure 2:**
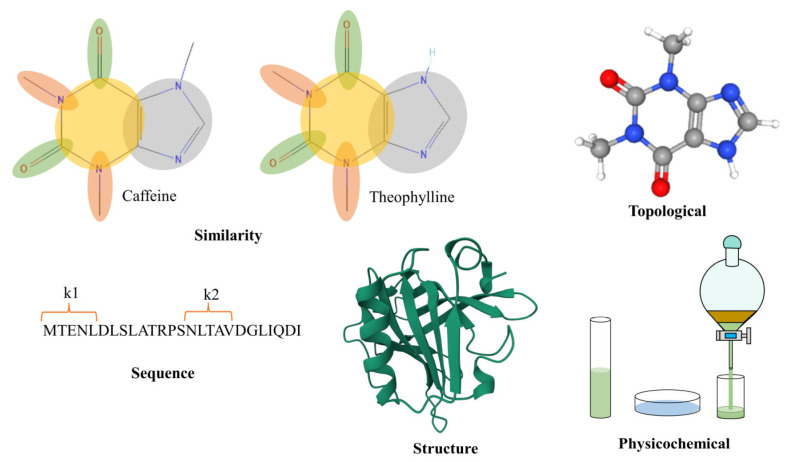
Visual representation of descriptors used in artificial intelligence.

When considering substrates, the approach used differs significantly from that employed for enzymes due to the disparity in size. However, common factors such as similarity, compound topology, and properties remain crucial in the analysis. Kroll used a topological approach based on fingerprints and properties to predict the value of the Michaelis constant in enzyme–substrate pairs, testing it in 47 model organisms and obtaining values similar to the original ones [49]. Yang compared the results achieved through similarity-based approaches and topology-based approaches, noting that they do not yet match experimental standards but show promise [51]. Additionally, Yang introduced a novel topology-based approach. Figure 2 shows graphically an example of each of the categories of molecular descriptors mentioned above. If you want to delve deeper into molecular descriptors, the Supplementary Material accompanying this article is available for your consultation.

## 5. Artificial Intelligence Models

Artificial intelligence models are algorithms and structures that allow the simulation of learning and performing specific tasks [8,12]. These are the basis of many machine learning systems and can be applied in a wide variety of domains, such as image pattern recognition, natural language processing, and decision making [52]. Often, these models are represented by formulas and operations that capture the underlying relationships and patterns in the data [9].

Artificial intelligence can be divided into two main areas: conventional artificial intelligence, which refers to techniques used before their widespread adoption in computer systems, and machine learning, which enables a system to learn from data rather than relying on explicit programming. Machine learning is subdivided into three main branches: supervised learning, unsupervised learning, and reinforcement learning, each with its own distinctive characteristics. Reinforcement learning involves a system learning through trial and error, where it is given a reward if it produces the correct result and a penalty if it does not. Unsupervised learning refers to teaching a system when labeled data are not available, and it is generally used to give a meaningful structure to the database. Lastly, supervised learning involves using labeled data to train a system.

**Figure 3:**
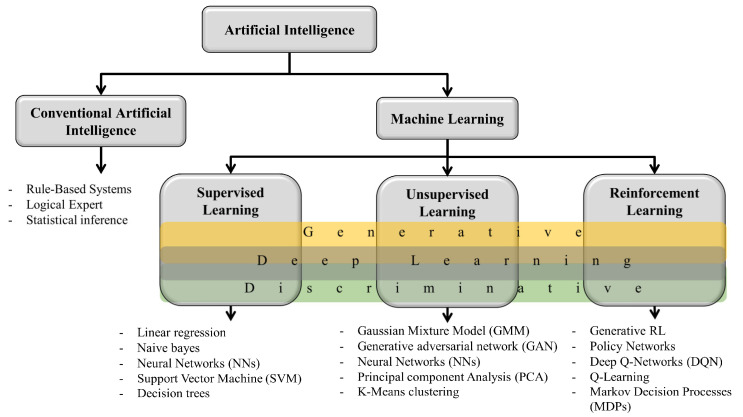
Classification of artificial intelligence and methods used in each type.

The algorithms used in these three branches of machine learning can be classified as discriminative and generative: discriminative methods focus on classifying data based on category differences, while generative methods model the distribution of data and can create new samples [52]. Figure 3 shows the division of artificial intelligence graphically. In the prediction of enzyme–substrate interactions, the most used algorithms are discriminative, since there exist limited training data [5]. Among the discriminative methods most used in the prediction of enzyme–substrate interactions, we find the algorithms based on support vector machines, neural networks, and decision trees [53]. Table 3 shows some enzyme-substrate interaction studies that have been conducted in recent years, specifying the algorithm used, the application, and the performance obtained.

### 5.1. Support Vector Machine (SVM) Models

**Figure 4:**
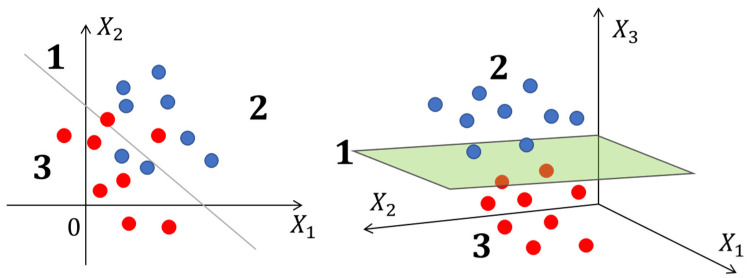
Data separation in a support vector machine for 2 and 3 dimensions (left and right respectively). The red and blue circles and spheres represent the training data, with the red ones being negative data and the blue ones being positive data. The data separation is performed using a line (in 2D) or a plane (in 3D). In region 2, positive data is located, and in region 3, negative data is located.

SVMs are algorithms widely used in artificial intelligence (AI) for classification. These models are based on the idea of finding the optimal hyperplane that separates the data into different classes or fits the best possible line for a separation of target (positive) and non-target (negative) class data [9]. Figure 4 graphically shows the separation of classes in an SVM. SVMs have been proven to be effective in analyzing large amounts of data for complex problem solving and decision making [8,17,44].

SVMs can address the inherent complexity of molecular data, making it possible to work with high-dimensional and non-linear data sets. This is crucial, as the relationships between chemical compounds can be intricate and difficult to capture with simpler methods. By being able to map the data to a higher dimensional feature space using kernel functions, SVMs can capture non-linear patterns and improve the accuracy of predictions [44,62,63].

The main advantage of these models is that they offer a clear visual representation of the separating hyperplane and support vectors, which makes it easier to interpret the results and understand the relationships between variables and classes. This interpretability is fundamental for the elucidation of synthesis routes, since it allows us to identify relevant molecular characteristics and understand how they influence enzyme–substrate interactions.

This type of model has been used in the prediction of thioesterase enzymes with acyl-ACP substrates, having mean accuracy rates of 80% with a deviation of 0.09, finding that the most relevant characteristics were Spectrum, Gappy, CKSAAP, KSCTriad, Moran, and similarity [5].

In their article, Ben-Hur et al. highlight the value of support vector machines and kernel techniques in the analysis and processing of complex biological data, proving their importance in predicting computational structures, proteins, the classification of genetic sequences, and the identification of functional regions in genomes, obtaining accuracies greater than 77% in the three cases [44].

In another study, two-layer SVM classifiers were used to improve remote protein homology detection and fold recognition. This work has had a considerable impact on the understanding of protein evolution and function [64].

Machine learning, particularly the SVM algorithm, has shown the capability to distinguish enzymatic and non-enzymatic metals in proteins with high precision [62]. This method offers a valuable tool for identifying and characterizing metals in proteins, potentially contributing significantly to the understanding of biological mechanisms and designing new therapies and enzymes.

These studies highlight how the use of SVMs in biology is driving significant advances in our understanding and ability to address complex challenges. If you wish to delve deeper into support vector machine algorithms, the Supplementary Material accompanying this article is available for your consultation.

### 5.2. Neural Network Models

**Figure 5:**
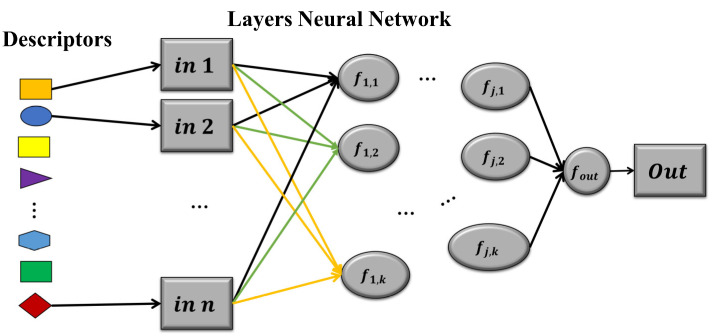
Model of a neural network with n descriptors as inputs, j layers, k neurons per layer, and one output. The geometric shapes and colors represent the different molecular descriptors and features that enter the model. The arrows indicate the connections or dependencies, also representing how information flows in the network.

Neural network models are one of the fundamental pillars of modern artificial intelligence (AI). These models are inspired by the structure and functioning of the human brain and are used to solve a wide range of problems, from image recognition to natural language processing [8]. Neural networks are made up of multiple layers of interconnected artificial neurons, which work together to process information and perform machine learning tasks [65,66]. Figure 5 shows a neural network model that uses molecular descriptors as inputs and has as output the presence or absence of interaction between an enzyme and a substrate.

The use of neural networks to elucidate synthetic pathways is highly beneficial due to their ability to learn complex and non-linear patterns in molecular data sets, handling high-dimensional data [8]. Neural networks offer a powerful tool for the prediction and optimization of synthesis pathways and have numerous advantages in this context.

One of the main advantages of neural networks is their ability to model non-linear relationships between molecular features and properties of interest. Synthesis pathways involve a series of complex molecular interactions and transformations, and neural networks are capable of capturing and learning these subtle and non-linear patterns [26,54]. This allows them to discover hidden connections and relationships that might go unnoticed with simpler or linear approaches; a major drawback is that we lose data interpretability.

These approaches have been primarily utilized for the classification of enzymes, substrates, and sequences, delivering results with accuracies exceeding 85%. Furthermore, they have been employed in the prediction of enzyme–substrate interactions [4,5,7,20,54,55].

Goldman et al. employed neural networks in their approach to model the specificity of enzymes and substrates at the family level. This involved capturing complex relationships between the characteristics of enzymes and substrates and assessing their impact on specificity [56]. Additionally, Li et al. utilized a multi-objective network-based approach to predict interactions, resulting in more accurate and comprehensive predictions [67]. These efforts showcase the diverse applications of neural networks in the study of enzyme–substrate specificity and interaction prediction.

Another application of neural networks in proteins is the prediction of structures and their interactions. Baek et al. developed neural networks with three tracks: amino acid sequence, conserved evolutionary information, and interaction profiles. This approach allows for the use of multiple sources of information and the capture of complex protein features [57].

Convolutional networks are also used for the prediction of enzyme–substrate interactions, as shown by Upadhyay et al. [64]. In their article, they focused on the use of convolutional neural networks to classify and re-engineer known enzymes in order to achieve new substrate activities [64]. If you wish to delve deeper into neural network algorithms, the Supplementary Material accompanying this article is available for your consultation.

### 5.3. Decision Tree Models

Decision tree models are a powerful technique used in AI to make decisions and perform classification and regression tasks. These models are based on the idea of constructing a decision tree that represents a series of questions and conditions about the input data’s characteristics. The tree uses these questions to arrive at a conclusion or prediction, making it a technique rooted in logic and reasoning [9,17].

Decision trees are a promising option for the elucidation of synthesis routes due to their clear and understandable interpretation, their ability to handle different types of data, their efficiency in handling large data sets, their ability to capture non-linear relationships, and their adaptability to imbalanced data situations [17].

**Figure 6:**
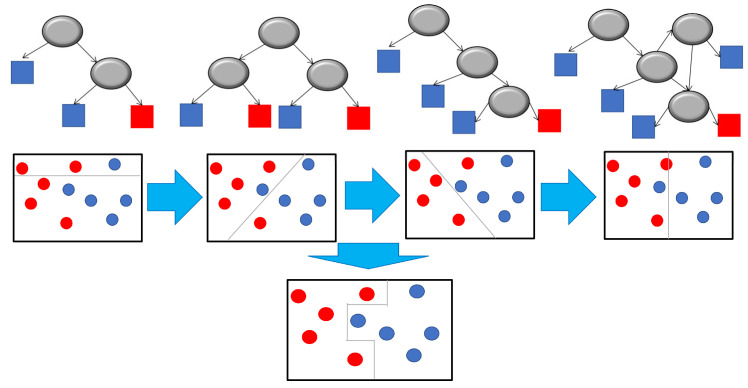
Gradient boosting tree built from four decision trees. Each tree tries to minimize the prediction error of the previous one. The circles symbolize the questions asked in the tree, the black arrows indicate how the information is propagated according to the response, the red and blue boxes indicate the classes to which they are classified according to the responses. The horizontal blue arrows represent the seriality of the trees, and the vertical ones the combination of all to achieve a more robust model. In this model, the aim is discriminate between the blue and red data.

These types of models have been used to predict properties of substrates and classify proteins; it is worth mentioning that there are more specialized methods such as gradient augmentation trees, these use several decision trees in sequence, where each one tries to minimize the prediction error of the previous one. [5,59,60,61]. Figure 6 shows a gradient boosting tree made up of four trees.

Costa et al., in their research, used decision trees to compare different approaches in hierarchical protein classification [59]. Also, in their research, Ebrahimi et al. focused on the use of decision trees to predict the thermostability of enzymes based on the amino acid sequence [60].

Banerjee et al. introduced a tool called “EnZymClass” which uses gradient boosting decision tree learning to predict the substrate specificity of acyl-ACP thioesterases in plants [5]. This tool demonstrates high prediction accuracy and has the potential to be a valuable contribution to plant enzyme research and design.

Additionally, the same author makes use of decision trees, neural networks, and support vector machines to predict the activity and substrate specificity of OleA enzymes, providing a valuable tool for engineering enzymes in the β-pathway oxidation of fatty acids [7].

If you wish to delve deeper into decision tree models, the Supplementary Material accompanying this article is available for your consultation.

### 5.4. Other Models

In addition to discriminative models of supervised learning, more complex artificial intelligence models have been used for predicting enzyme–substrate interactions, such as convolutional networks and deep learning, showing performances exceeding 70%, even with substrates dissimilar to those in training (similarity <40%) [20,58]. These methods have been employed in general models to predict enzyme–substrate activity, leveraging larger data sets compared to the approach of modeling a single reaction.

Machine learning diffusion models have been recently used alongside generative models of deep learning to explore the latent space of a set of enzymes or substrates with a common function, thus creating new enzymes with higher affinity to a substrate or generating ligands with high specificity and affinity for target proteins [68,69,70,71]. This novel approach has been gaining prominence among supervised learning methods, as it theoretically allows us to find the useful space of the complex without the use of descriptors, providing a better understanding of the system to the model.

## 6. Model Validation

The validation of artificial intelligence models is a crucial process to evaluate the performance and predictive capacity of a model [8]. This process involves measuring how the model performs on unseen data, i.e., data that were not used during the model training. The goal is to determine if the model is capable of generalizing well to new data and is not overfitting or underfitting [9]. Overfitting refers to when a model does not fit the generality of the training data but instead memorizes or learns them in a very specific way. Underfitting refers to when a model fails to learn important relationships and features from the training data.

There are different model validation techniques used in machine learning. The most common one is cross-validation, where the data are split into training (which includes both training and validation) and testing sets.

### 6.1. Cross-Validation

Cross-validation is a technique that allows for the estimation of model performance using the entire available data set. In this technique, the data set is divided into two sets: one for model training and the other for model evaluation or testing. Typically, a ratio of 70–80% for training and 20–30% for testing is used, although this can vary depending on the size of the available data set [9,13]. The training data are divided into k subsets or folds of approximately the same size. Then, an iterative process is performed where the model is trained on k-1 folds and evaluated on the remaining fold (usually called validation). This process is repeated k times, ensuring that each fold is used as a validation set once.

### 6.2. Out-of-Sample Validation

Out-of-sample validation is a fundamental technique in the evaluation of ML models that facilitates the assessment of their performance with data not seen during training and validation. In this methodology, the data set is divided into two parts: one is used for training and validation, and the other is reserved for testing. This practice ensures that the model has not been previously exposed to the test data, allowing it to confront new information and evaluating its ability to generalize [72].

The typical data split is 80–90% for training and validation and 10–20% for testing. This is a sufficient amount of data for learning and validation without compromising the model’s ability to generalize to new instances.

Additionally, the technique can be complemented with cross-validation in the training and validation set. This provides a more robust evaluation and reduces the impact of the initial data split choice.

### 6.3. Experimental Validation

Experimental validation involves the laboratory confirmation of predicted enzyme–substrate activities, as exemplified Banerjee et al. [5]. This process entails inducing the protein and measuring its activity in the presence of the substrate. Although it is a robust validation, it is inefficient when dealing with extensive validation data sets due to the high costs, intensive labor, and time required.

Amin et al. made predictions regarding three oxidoreductase enzymes with the capacity to convert myo-inositol to scyllo-inosose by reducing NAD+. Subsequently, one of these enzymes, dhaf_2064, was experimentally evaluated, demonstrating activity towards the substrate and validating the initial prediction [19].

Repečka et al. used generative adversarial networks to generate sequences of malate dehydrogenases, successfully obtaining 60 artificial proteins. These proteins were experimentally tested, revealing that 13 of them exhibited activity similar to natural variants [73].

On the other hand, Banerjee et al. used EnZymClass to predict three enzyme sequences encoding medium-chain thioesterases of the TE14 family. These predictions underwent experimental testing, confirming that two of the sequences exhibit activity with the substrate [5].

In these cases, the combination of computational predictions and experimental validation demonstrates the usefulness of these tools in the identification and characterization of enzymes with specific activities, significantly contributing to the advancement in understanding metabolic pathways and molecular biology.

To measure how a discriminative algorithm behaves with the training data, there are different metrics such as accuracy, sensitivity, specificity, precision, F1 score, AUROC, and AUPR.

*Accuracy* refers to the number of successes that the model had when classifying a piece of data. The basic formula for calculating precision in cross-validation is as follows:

(1) Accuracy=(TP+TN) / (TP+TN+FP+FN) 

In this formula, TP represents true positives, TN true negatives, FP false positives, and FN false negatives. Accuracy is a measure of how well the model can correctly predict positive and negative instances.

*Sensitivity* measures how well the model can identify cases labeled as positive. It is calculated using the following formula:

(2) Sensitivity=(TP) / (TP+FN) 

*Specificity* measures how well the model can identify cases labeled as negative. It is calculated using the following formula:

(3) Specificity=(TN) / (TN+FP) 

*Precision* measures the proportion of positive predictions delivered by the model. It is calculated using the following formula:

(4) $$precision=\frac{TP}{TP+FP}\;$$

The *F*1 *score* combines sensitivity or recall and precision through a harmonic mean [74]. It is calculated using the following formula:

(5) $$F1\;score=2\frac{sensitivity\times precision}{sensitivity+precision}\;$$

The *AUROC*, the area under the ROC (Receiver Operating Characteristic) curve, allows us to observe the false positive rate and the false negative rate, providing a general measure of the model’s performance [74].

The *AUPR* (Area Under the Precision–Recall Curve) allows us to observe precision and recall (sensitivity) in a 2D graph, offering a better overall view than the AUROC when the data set is unbalanced.

This is the most commonly used technique among the many model validation methods used in artificial intelligence. It is important to select the appropriate technique based on the type of problem and the available data. Additionally, it is recommended to perform multiple validation iterations using different techniques or data splits to obtain a more robust evaluation of the model.

## 7. Limitations, Challenges, and Conclusions

Although the application of AI in the prediction of enzyme–substrate interactions for the elucidation of synthesis pathways has shown promising results, there are certain limitations and challenges that need to be addressed to ensure the reliability and applicability of AI models, such as the availability of reliable data, the interpretability of the results, generalization of the AI models, the complexity of the interaction, the few experimental data, and the low capacity for experimental validation of results.

To train accurate AI models, a large, well-annotated, high-quality data set is required that contains information about enzyme amino acid sequences, three-dimensional structures, and the properties of substrates. The availability of this data can be challenging, as collecting and curating complete and reliable data sets can be expensive and time-consuming. In addition, the lack of uniformity in data annotation and standardization in databases complicates the comparison and integration of different data sets, increasing development time due to data curation, validation, and homogenization and limiting the development of general models.

Another limitation is that AI models, such as neural networks, are often considered “black boxes” due to the complexity of their algorithms. This makes it difficult to interpret the results and understand the factors that influence the predictions. Interpreting the results of AI models and explaining how a certain prediction was arrived at are major challenges in the field. The ability to understand and explain AI predictions is crucial to gain the trust of the scientific community and facilitate informed decision making in elucidating synthetic pathways.

AI models may show optimal performance on training data but may have difficulty generalizing and adapting to new situations or unseen data. The ability to transfer learned insights from one data set to another is a major challenge in the field of AI. The lack of generalization may limit the applicability of the models in the prediction of the enzyme–substrate interaction in different contexts and biological systems.

Enzyme–substrate interactions can be influenced by a variety of factors, such as pH, temperature, and the presence of cofactors. However, the data sets used to train AI models often do not fully capture this variability, which can affect the models’ ability to predict interactions under different experimental conditions.

Although experimental data are essential for training and validating AI models, sometimes the availability of this data is limited. This can make it difficult to develop accurate and reliable models, especially in cases where enzyme–substrate interactions are poorly studied or understood. Increasing the availability of experimental data in databases is necessary to improve the quality and quantity of data sets used in AI models. Additionally, the experimental validation of predictions will help focus AI algorithms on the problem, allowing them to better capture the system’s dynamics. However, this is a slow and costly process, limiting progress.

As for future prospects, it is crucial to continue researching and developing new AI techniques and approaches to improve the prediction of enzyme–substrate interactions in pathway elucidation. Game theory, natural language processing, deep learning, and reinforcement learning perspectives can be applied. This requires collaboration among researchers in different disciplines, such as biology, bioinformatics, and artificial intelligence, to address existing challenges and overcome current limitations.

In conclusion, AI offers great potential for accelerating and improving the elucidation of synthetic pathways through the prediction of enzyme–substrate interactions. Although there are limitations and challenges, the use of AI in this field remains promising. With proper attention to these challenges and continuous research, AI has the potential to revolutionize the way we understand and manipulate metabolic pathways for the production of industrial, pharmaceutical, and agricultural compounds of interest.

## Figures and Tables

**Table 1:** Summary of databases that have enzyme information.

Database	Type of Information	Quantity of Proteins	Main Focus	Strengths	Weaknesses	Ref.
UniProt	Sequences, functions, and structures	248,272,897 structures (569,793 reviewed)	Proteins and their attributes	Wide coverage and comprehensive and updated information	Redundant and unreviewed data	[30,31]
PDB	Structural information	208,066 PDB structures + 1,068,577 computerized structure models	3D structures of proteins and enzymes	Revised and non-redundant database	Focus only on structure	[32]
BRENDA	Functional and metabolic information	32,832,265 sequences, 90,000 enzymes, and 13,000 organisms	Enzymes, their reactions, and biochemical properties	Database specialized in enzymes, their function, biochemical properties, and reactions; revised database	Slow updates; requires prior knowledge in biochemistry and molecular biology	[34]
KEGG	Information on metabolic pathways and gene/protein functions	1,098,631 metabolic pathways and 49,962,693 genes	Metabolic pathways and gene functions	Interconnection with other databases	Requires prior knowledge in biochemistry and molecular biology	[35]
NCBI	Protein sequences, structures, gene sequences, and annotations	40,000,000	Various protein information	Interconnection with other databases	Redundant information	[36]
MetaCyc	Metabolic pathways and enzymes	>2749 pathways	Metabolic pathways and enzymes from different organisms	Revised and non-redundant database	It is limited to metabolic pathways and enzymes	[33,37]

**Table 2:** Summary table of studies using different types of descriptors. The table shows which descriptor categories are most commonly used for enzymes and substrates and highlights examples of specific approaches.

Main Approach	Type of Descriptors	Data Used	References
Prediction of range of substrates in bacterial nitrilases	Sequence, physicochemical, and structure	Experimental activity data, alignments, electrostatic potential, and 3D substrate structure	[18]
Detection of functional similarities	Sequence and structure	Alignments, sequences, and structures	[19]
Discrimination of substrate function	Sequence and structure	Alignments and fingerprints	[45,46]
Approach based on fingerprints and properties: Michaelis constant prediction	Similarity and topology	Fingerprints, molecular weight, LogP, and others	[49]
Comparison of results and new approaches	Similarity and topology	Fingerprints and MPNN	[29]

**Table 3:** Machine learning algorithms in biological research. This table provides an overview of machine learning algorithms used in various biological research applications. It includes the name of the algorithm, its application, performance, and the relevant reference.

Algorithm	Application	Performance	References
Support vector machines (SVMs)	Enzymatic and substrate classification and prediction	80% accuracy	[5]
Support vector machines (SVMs) and kernel techniques	Analysis and processing of complex biological data	77–91.4% accuracy	[44]
Decision trees	Differentiation of metals in proteins	94.2% accuracy	[6]
Neural networks	Classification of enzymes, substrates, and sequences	>85% accuracy	[4,54,55]
Neural networks	Prediction of enzyme–substrate interactions	>73.2%	[5,7,20]
Neural networks	Prediction of enzyme specificity	Complex relationship capture	[56]
Neural networks	Prediction of protein structures and interactions	Capture of complex features	[57]
Convolutional neural networks	Enzyme classification and redesign	80.72% accuracy	[58]
Decision trees	Protein classification and regression	62%	[59,60]
Gradient augmentation trees	Prediction of enzyme activity	79%	[5,61]
Random forest, feedforward neural network, and Naive Bayes	Prediction of OleA enzyme activity and specificity	82.6%, 73.2%, 58.6%	[7]

## Data Availability

No data were used for the research described in this article.

## Supplementary Materials

The following supporting information can be downloaded at: https://www.mdpi.com/article/10.3390/metabo14030154/s1. Figure S1: Graph of equations 2, 3, and 4; green line represents equation 2, blue region represents equation 3, and orange region represents equation 4; Figure S2: Graph of equations 2, 3 and 4 next to the training data; Figure S3: neural network with n inputs, one neuron, one layer and a single output; Figure S4: neural network with n inputs, m neurons, two layers (one input and one output) and a single output; Figure S5: neural network with n inputs, m+l+…+i neurons, k+1 layers and a single output; Figure S6: neural network with n inputs, m+l+…+i+j neurons, k+1 layers and j outputs; Figure S7: left, decision tree of three conditions and two possible outputs (red or blue). Right, clustering performed by the decision tree from the training data.
