# Supplementary material for: Machine Learning to Predict Enzyme–Substrate Interactions in Elucidation of Synthesis Pathways: A Review
Source: Metabolites. 2024 Mar 7;14(3):154. doi: 10.3390/metabo14030154 (PMC10972002; doi:10.3390/metabo14030154)
Supplement: Supplementary file 1 [file metabolites-14-00154-s001.zip › metabolites-2850967-supplementary.pdf]

Supplementary Material

Machine learning to predict enzyme-substrate interactions in elucidation of synthesis pathways: A review

1 Molecular Descriptors

Molecular descriptors are numerical representations or mathematical encodings that capture information about the properties and characteristics of a molecule in the fields of chemistry and bioinformatics.

Molecular descriptors are numerical or symbolic representations of molecules used to describe their structural, chemical, or physical characteristics [1,2]. In the context of enzymes and substrates, molecular descriptors play a crucial role in predicting enzyme-substrate interactions and elucidating synthetic pathways by capturing different aspects of enzymes and substrates, providing valuable information for understanding their features and properties. These descriptors are used to summarize the structure and properties of a molecule in a way that can be computationally analyzed and compared. They may include chemical composition, molecular geometry, physical and chemical properties, atom connectivity, electrical charges, polarity, functional groups, and many other relevant features.

Descriptors can be grouped into five types: molecular structure, amino acid sequence, physicochemical, molecular topology, and similarity [3]. Sequence and structure-based descriptors are the most commonly used for enzymes [3–10], utilizing detailed information about protein composition and configuration. On the other hand, similarity, topology, and physicochemical descriptors are most commonly used for substrates [3,11–15]allowing the evaluation of how molecules participating in enzymatic reactions interact and behave.

Structure

- 3D structure
- Superficial area
- Ring Size
- Turning radius
- Molecular diameter
- Surface Area

Sequence

- Sequence Length
- Composition Amino Acid
- Frequency Amino Acid
- Hydrophobic Amino Acid Composition
- Sequential Motif Frequency
- Tripeptide

- Bipeptide
- Domain Length
- Kmers
- Kernels

Physicochemical

- Melting Point
- Boiling Point
- Density
- Solubility
- Molecular weight
- LogP
- Viscosity
- Refractive Index
- Electrical Conductivity

Topology

- Connectivity Index
- Wiener Index
- Number of Atoms
- Number of Bonds
- Randic Index
- Number of Carbon Atoms
- Number of Hydrogen Atoms
- Fingerprints

Similarity

- Tanimoto Coefficient
- Tversky Similarity
- Dice-Bray-Curtis Coefficient
- Jaccard Coefficient
- Kulczynski Coefficient
- Euclidean Distance

2 Machine Learning Methods

2.1 Support Vector Machine

Support Vector Machines (SVMs) are widely used algorithms in artificial intelligence (AI) for classification and regression. These models are based on the idea of finding the optimal hyperplane that separates data into different classes or fits the best possible line for regression. SVMs have proven to be effective in solving complex problems and making decisions [4,16,17].

The basic formulation of an SVM model for linear classification can be expressed as follows:

$$y(x) = sign(w^T x + b) \quad (ecc1)$$

In this formula, x represents the feature vector of an input instance, w is the weight vector defining the optimal hyperplane, and "b" is the bias or intersection term. The $sign()$ function assigns a specific class to the input instance, meaning if the formula's result is positive, it is assigned to one class, and if it's negative, it's assigned to another class. Let's expand equation 1 for 2 and 3 inputs, which in our case will be the molecular descriptors. For 2 inputs, we have:

$$sign\left(\begin{bmatrix} w_1 \\ w_2 \end{bmatrix} [x_1 \quad x_2] + b\right) = sign(w_1 x_1 + w_2 x_2 + b)$$

Now we apply the sign function, which will give us two categories: one when the function gives us a value less than zero and another when it's greater than zero. If it's equal to zero, it corresponds to the line that divides both. We apply it, and we have:

$$b + w_1 x_1 + w_2 x_2 = 0 \quad (ecc2)$$

$$b + w_1 x_1 + w_2 x_2 > 0 \quad (ecc3)$$

$$b + w_1 x_1 + w_2 x_2 < 0 \quad (ecc4)$$

When graphing equations 2, 3, and 4, we obtain Figure S1, where two regions can be distinguished. The blue region represents equation 3, the orange region represents equation 4, and the green line is equation 2, the line that separates both regions. This division will allow us to classify data into two categories.

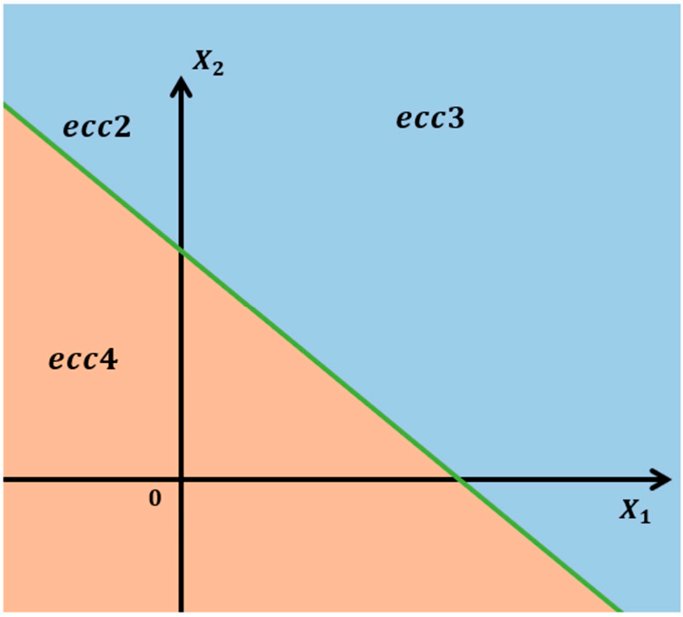

Figure S1, *Graph of equations 2, 3, and 4; green line represents equation 2, blue region represents equation 3, and orange region represents equation 4.*

To make the SVM model work optimally, it's necessary to find the appropriate values for "w" and "b." This is achieved through the training process, which involves maximizing the distance between the

hyperplane and the closest data points to it, known as support vectors. The goal is to find the hyperplane that maximizes the margin between classes, ensuring good generalization and better performance on previously unseen data. To find these values, we apply the following equation:

$$max\frac{1}{2}\|W\|^2$$

Subject to:

$$y_i(Wx_i + b) \geq 1 \ \forall \ i$$

Where i represents the training data and y_i the category to which the training data belongs. When we apply it to the two descriptors, we get:

$$max\frac{1}{2}(w_1^2 + w_2^2)$$

Subject to:

$$y_i(w_1x_{1i} + w_2x_{2i} + b) \geq 1 \ \forall \ i$$

By applying the training data to maximization and graphing it with equations 2, 3 and 4 we have:

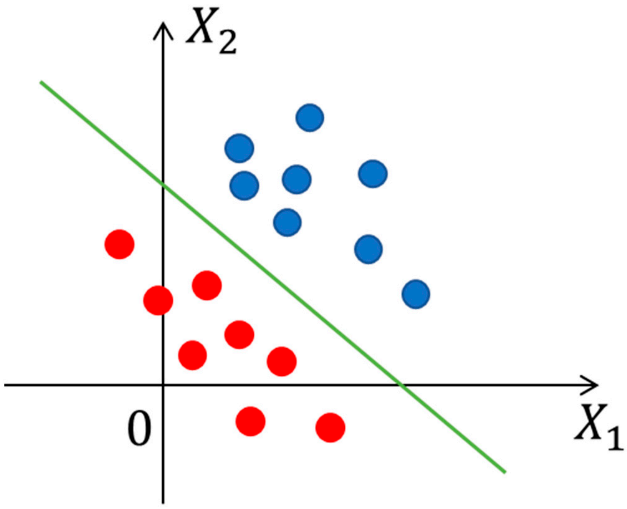

***Figure S2**, Graph of equations 2, 3 and 4 next to the training data.*

2.2 Neural network models

Neural network models are one of the fundamental pillars of modern artificial intelligence (AI). These models are inspired by the structure and functioning of the human brain and are used to solve a wide range of problems, from image recognition to natural language processing. Neural networks are composed of multiple layers of interconnected artificial neurons, which work together to process information and perform machine learning tasks [13].

The basic formula for calculating the output of a neuron in a neural network can be expressed as:

$$y = f(\Sigma(w * x) + b)$$

In this formula, y represents the output of the neuron, w are the synaptic weights that determine the importance of the connections between the neuron and the neurons of the previous layer, x are the inputs to the neuron and b is the bias or intersection term. The function $f()$ is the activation function that introduces nonlinearity in the neural network.

There are different types of activation functions used in neural networks. Some of the most common are:

Sigmoid function: $f(x) = 1 / (1 + e^{-x})$

ReLU (Rectified Linear Unit) function: $f(x) = max(0, x)$

Tanh Function (Hyperbolic Tangent): $f(x) = (e^{x} - e^{-x}) / (e^{x} + e^{-x})$

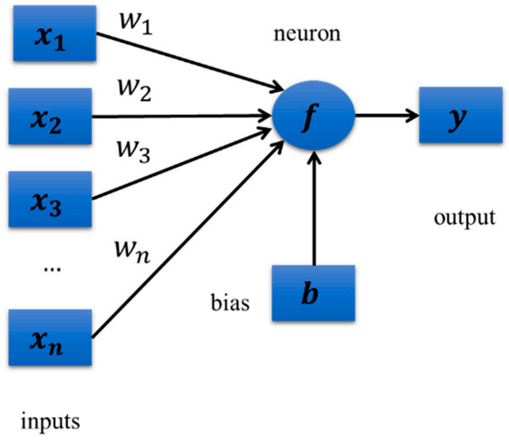

***Figure S3**, neural network with n inputs, one neuron, one layer and a single output*

Figure S3 shows a neural network with one input, one layer, n inputs and one output, but we can also have several neurons in a layer (figure S4). If we apply the outputs of one layer to another, we will have a multilayer network, that is, the previous layer becomes the input of the neurons of the next layer (figure S5). This allows information to flow through the neural network and calculations and transformations to be performed at each layer to extract features and learn useful representations of the data, we can also have multiple outputs (figure S6).

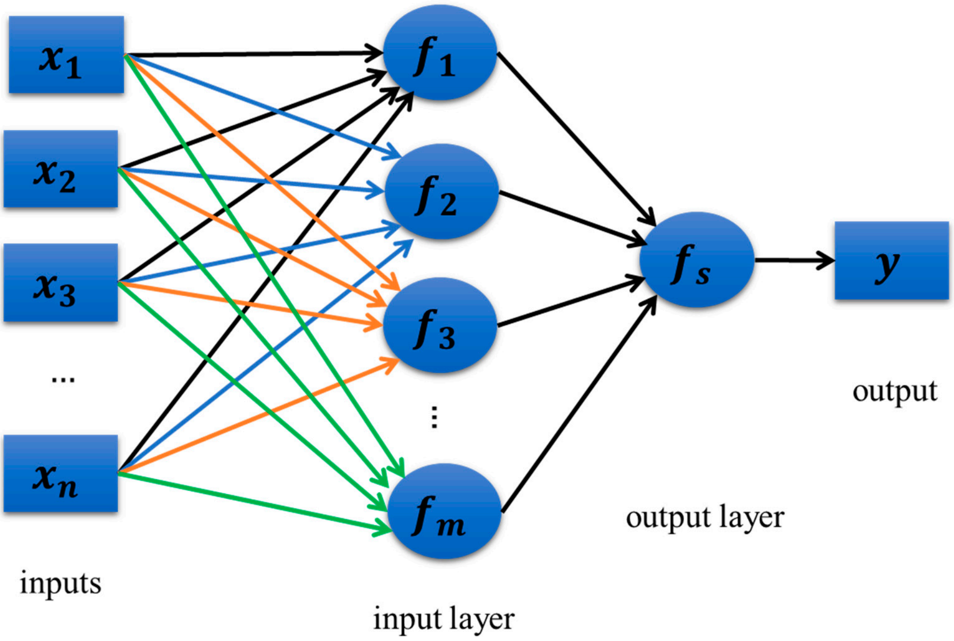

Figure S4, *neural network with n inputs, m neurons, two layers (one input and one output) and a single output*

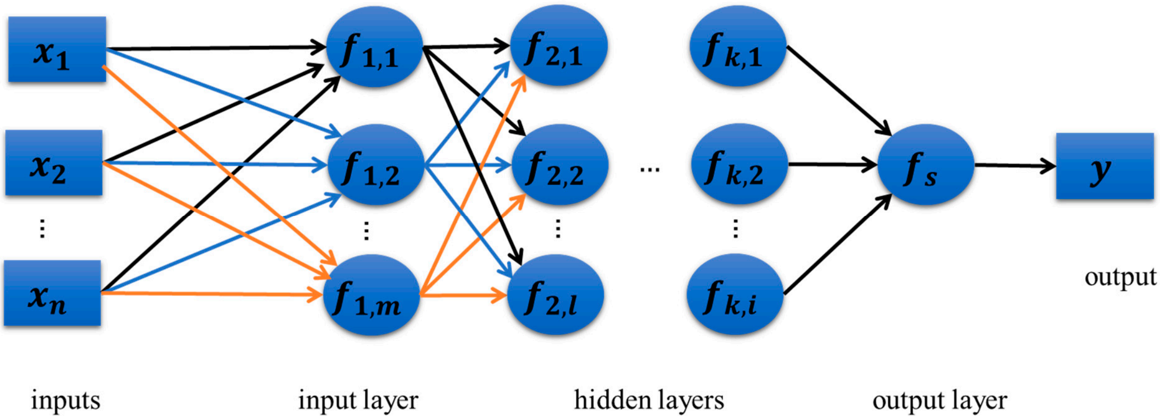

Figure S5, *neural network with n inputs, m+l+…+i neurons, k+1 layers and a single output*

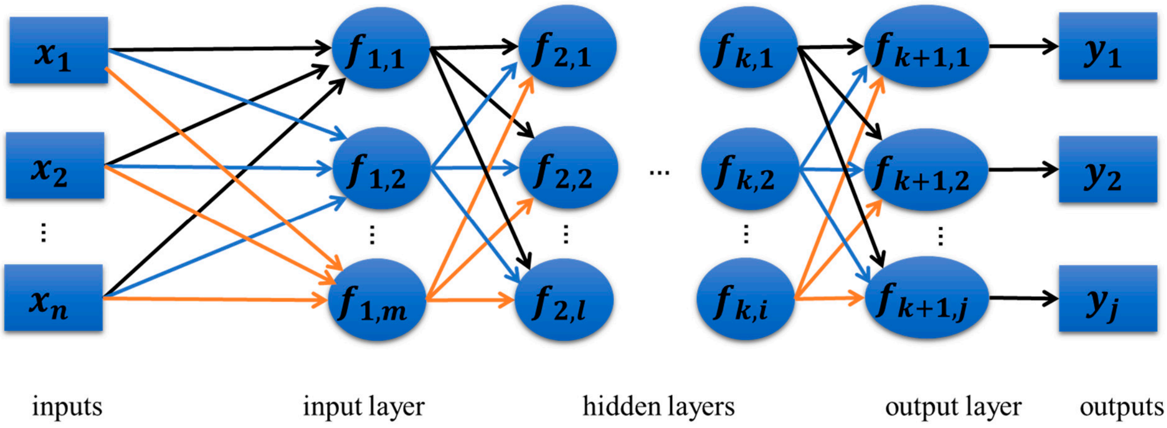

***Figure S6**, neural network with n inputs, m+l+…+i+j neurons, k+1 layers and j outputs*

One of the most used architectures in neural networks is the deep neural network, also known as a neural network with multiple hidden layers. The hidden layers are the intermediate layers of the network. The formula for calculating the output of a layer in a deep neural network can be expressed as:

$$y = f(W * x + b)$$

Here, W is the weight matrix that connects the neurons of one layer with those of the next layer, x is the input vector and b is the bias vector. The $f()$ function is applied to each element of the result

In the training stage of a neural network, weights and biases are iteratively adjusted using optimization algorithms, such as gradient descent or Adam (adaptive moment estimation), to minimize a loss function that measures the discrepancy between the outputs. predicted and actual values. This allows the neural network to learn to make more accurate predictions as it is presented with a training data set.

Another important concept in neural networks is that of recurrent connections, which allow information to flow in loops through the network. This is especially useful in sequential processing applications, such as time series analysis or natural language processing. Recurrent neural networks (RNN) use recurrent connections to capture long-term dependencies in data.

2.3 Decision tree models

Decision tree models are a powerful technique used in artificial intelligence (AI) to make decisions and perform classification and regression tasks. These models are based on the idea of building a decision tree that represents a series of questions and conditions about the characteristics of the input data, and uses those questions to reach a conclusion or prediction [9,17–19].

The basic formula for a decision tree is as follows:

```
if (condition1) then (action1)

else if (condition2) then (action2)
```

```
...

else (actionN)
```

In this formula, `condition1, condition2, ..., conditionN` are the questions or conditions about the characteristics of the data, and `action1, "action2", ..., actionN` are the actions or decisions that are taken based on the answers to those questions. Each condition in the tree is based on a specific characteristic of the input data, and the branches of the tree represent different possible values for that characteristic.

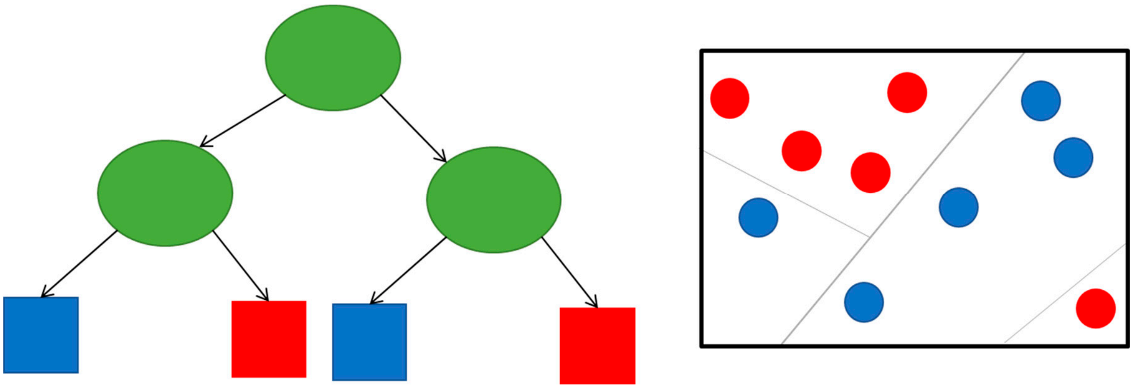

Figure S7*, left, decision tree of three conditions and two possible outputs (red or blue). Right, clustering performed by the decision tree from the training data*

Decision trees are built using algorithms that seek to divide the data set into more homogeneous subsets in terms of the target variable to be predicted. One of the most widely used techniques for constructing decision trees is the classification and regression tree (CART) algorithm. The CART algorithm uses Gini impurity or entropy as measures to evaluate the quality of a split and choose the best question or condition at each step.

The Gini impurity can be calculated with the following formula:

$$Gini(p) = 1 - \Sigma (p_i)^2$$

In this formula, p is a vector that represents the proportion of each class in a subset of data. The term $\Sigma (p_i)^2$ represents the sum of the squares of those proportions. A low Gini impurity value indicates that a subset is purer and contains a single class.

Entropy is calculated using the formula:

$$Entropy(p) = -\Sigma\, p_i * \log_2(p_i)$$

Here, p_i represents the proportion of each class in the data subset. Entropy measures the uncertainty in the data set. A low entropy value indicates that a subset is more homogeneous and contains a single class.

Once the decision tree is built, it is used to make predictions on new data by following the path from the root to the leaves of the tree, answering the questions at each node and making the corresponding

decisions. Depending on the task, decision trees can also be used to calculate probabilities or perform regressions.

One of the challenges of decision trees is the tendency to overfit the training data and not generalize well to new data. To mitigate this problem, techniques such as tree pruning are used, which simplifies the tree by removing nodes and reducing its complexity.

References

1. Parthasarathi, R.; Dhawan, A. Chapter 5 - In Silico Approaches for Predictive Toxicology. In *In Vitro Toxicology*; Dhawan, A., Kwon, S., Eds.; Academic Press, 2018; pp. 91–109 ISBN 978-0-12-804667-8.

2. Chandrasekaran, B.; Abed, S.N.; Al-Attraqchi, O.; Kuche, K.; Tekade, R.K. Chapter 21 - Computer-Aided Prediction of Pharmacokinetic (ADMET) Properties. In *Dosage Form Design Parameters*; Tekade, R.K., Ed.; Academic Press, 2018; pp. 731–755 ISBN 978-0-12-814421-3.

3. Mou, Z.; Eakes, J.; Cooper, C.J.; Foster, C.M.; Standaert, R.F.; Podar, M.; Doktycz, M.J.; Parks, J.M. Machine Learning-Based Prediction of Enzyme Substrate Scope: Application to Bacterial Nitrilases. *Proteins: Structure, Function, and Bioinformatics* **2021**, *89*, 336–347, doi:https://doi.org/10.1002/prot.26019.

4. Ben-Hur, A.; Ong, C.S.; Sonnenburg, S.; Schölkopf, B.; Rätsch, G. Support Vector Machines and Kernels for Computational Biology. *PLoS Comput Biol* **2008**, *4*, e1000173-.

5. Yu, C.-Y.; Chou, L.-C.; Chang, D.T.-H. Predicting Protein-Protein Interactions in Unbalanced Data Using the Primary Structure of Proteins. *BMC Bioinformatics* **2010**, *11*, 167, doi:10.1186/1471-2105-11-167.

6. Saigo, H.; Vert, J.-P.; Ueda, N.; Akutsu, T. Protein Homology Detection Using String Alignment Kernels. *Bioinformatics* **2004**, *20*, 1682–1689, doi:10.1093/bioinformatics/bth141.

7. Amin, S.R.; Erdin, S.; Ward, R.M.; Lua, R.C.; Lichtarge, O. Prediction and Experimental Validation of Enzyme Substrate Specificity in Protein Structures. *Proceedings of the National Academy of Sciences* **2013**, *110*, E4195–E4202, doi:10.1073/pnas.1305162110.

8. Yang, K.K.; Wu, Z.; Arnold, F.H. Machine-Learning-Guided Directed Evolution for Protein Engineering. *Nat Methods* **2019**, *16*, 687–694, doi:10.1038/s41592-019-0496-6.

9. Çamoğlu, O.; Can, T.; Singh, A.K.; Wang, Y.-F. Decision Tree Based Information Integration for Automated Protein Classification. *J Bioinform Comput Biol* **2005**, *3*, 717–742, doi:10.1142/S0219720005001259.

10. Banerjee, D.; Jindra, M.A.; Linot, A.J.; Pfleger, B.F.; Maranas, C.D. EnZymClass: Substrate Specificity Prediction Tool of Plant Acyl-ACP Thioesterases Based on Ensemble Learning. *Curr Res Biotechnol* **2022**, *4*, 1–9, doi:https://doi.org/10.1016/j.crbiot.2021.12.002.

11. Kroll, A.; Ranjan, S.; Engqvist, M.K.M.; Lercher, M.J. A General Model to Predict Small Molecule Substrates of Enzymes Based on Machine and Deep Learning. *Nat Commun* **2023**, *14*, 2787, doi:10.1038/s41467-023-38347-2.

12. Kroll A.; Engqvist M.; Heckmann D.; Lercher M. Deep Learning Allows Genome-Scale Prediction of Michaelis Constants from Structural Features. *PLoS Biol* **2021**, *19*, 1–21, doi:10.1371/journal.pbio.3001402.

13. Prince, S.J.D. *Understanding Deep Learning*; The MIT Press, 2023; ISBN 9780262048644.

14. Taheri, K.; Moradi, H.; Tavassolipour, M. Collaboration Graph for Feature Set Partitioning in Data Classification. *Expert Syst Appl* **2023**, *213*, 118988, doi:https://doi.org/10.1016/j.eswa.2022.118988.

15. Zhou, J.; Cui, G.; Hu, S.; Zhang, Z.; Yang, C.; Liu, Z.; Wang, L.; Li, C.; Sun, M. Graph Neural Networks: A Review of Methods and Applications. *AI Open* **2020**, *1*, 57–81, doi:https://doi.org/10.1016/j.aiopen.2021.01.001.

16. Janiesch, C.; Zschech, P.; Heinrich, K. Machine Learning and Deep Learning. *Electronic Markets* **2021**, *31*, 685–695, doi:10.1007/s12525-021-00475-2.

17. Somvanshi, M.; Chavan, P.; Tambade, S.; Shinde, S. V A Review of Machine Learning Techniques Using Decision Tree and Support Vector Machine. In Proceedings of the 2016 International Conference on Computing Communication Control and automation (ICCUBEA); 2016; pp. 1–7.

18. Si, S.; Zhang, H.; Keerthi, S.S.; Mahajan, D.; Dhillon, I.S.; Hsieh, C.-J. Gradient Boosted Decision Trees for High Dimensional Sparse Output. In Proceedings of the Proceedings of the 34th International Conference on Machine Learning; Precup, D., Teh, Y.W., Eds.; PMLR, July 2017; Vol. 70, pp. 3182–3190.

19. Costa, E.P.; Lorena, A.C.; Carvalho, A.C.P.L.F.; Freitas, A.A.; Holden, N. Comparing Several Approaches for Hierarchical Classification of Proteins with Decision Trees. In Proceedings of the Advances in Bioinformatics and Computational Biology; Sagot, M.-F., Walter, M.E.M.T., Eds.; Springer Berlin Heidelberg: Berlin, Heidelberg, 2007; pp. 126–137.
